# Exploring candidate biological functions by Boolean Function Networks for *Saccharomyces cerevisiae*

**DOI:** 10.1371/journal.pone.0185475

**Published:** 2017-10-05

**Authors:** Maria Simak, Chen-Hsiang Yeang, Henry Horng-Shing Lu

**Affiliations:** 1 Bioinformatics Program, Taiwan International Graduate Program, Institute of Information Science, Academia Sinica, Taipei, Taiwan; 2 Institute of Statistics, National Chiao Tung University, Hsinchu, Taiwan; 3 Institute of Statistical Science, Academia Sinica, Taipei, Taiwan; 4 Big Data Research Center, National Chiao Tung University, Hsinchu, Taiwan; King’s College London, UNITED KINGDOM

## Abstract

The great amount of gene expression data has brought a big challenge for the discovery of Gene Regulatory Network (GRN). For network reconstruction and the investigation of regulatory relations, it is desirable to ensure directness of links between genes on a map, infer their directionality and explore candidate biological functions from high-throughput transcriptomic data. To address these problems, we introduce a Boolean Function Network (BFN) model based on techniques of hidden Markov model (HMM), likelihood ratio test and Boolean logic functions. BFN consists of two consecutive tests to establish links between pairs of genes and check their directness. We evaluate the performance of BFN through the application to *S*. *cerevisiae* time course data. BFN produces regulatory relations which show consistency with succession of cell cycle phases. Furthermore, it also improves sensitivity and specificity when compared with alternative methods of genetic network reverse engineering. Moreover, we demonstrate that BFN can provide proper resolution for GO enrichment of gene sets. Finally, the Boolean functions discovered by BFN can provide useful insights for the identification of control mechanisms of regulatory processes, which is the special advantage of the proposed approach. In combination with low computational complexity, BFN can serve as an efficient screening tool to reconstruct genes relations on the whole genome level. In addition, the BFN approach is also feasible to a wide range of time course datasets.

## Introduction

One of the challenging fields of computational biology is the study of gene regulatory networks (GRNs). The demanding task of recovering the hidden relations between genes at the whole-genome level can provide insights to the comprehensive understanding of biological pathways and their mechanisms. It can also enhance the developments for disease treatments and biological technology. Currently there are two major experimental approaches to identify regulatory relations between genes. The first type uses perturbation (knockout or overexpression) experiments to explore regulatory targets of specific gene. The second type detects targets for specific transcription factors (TFs) with protein-DNA binding experiments. Neither of two experimental techniques can be utilized to reconstruct GRN on genome-wide scale due to the demand of huge number of experiments. In addition, the abundant high-throughput observational transcriptomic data [1, 2, 3], including time course expression data, provides indirect evidence of gene regulatory networks.

To perform the task of gene regulatory network inference (GRNI) or reverse-engineering from available transcriptomic data, numerous computational methods from statistics and computer science were developed. Reviews of the existing approaches in GRN reverse-engineering [4, 5, 6, 7, 8] provide the comparisons of methods for a variety of categories: stochastic/deterministic, static/dynamic, discrete/continuous, bivariate/multivariate, linear/nonlinear, directed/undirected. Below we consider four major groups of methods which are used for GRN inference from time course data: Boolean networks, Bayesian Networks, information theory models and graph-based methods.

Boolean networks (BNs) [9, 10] and its stochastic extension to probabilistic Boolean networks (PBNs) [11, 12] are discrete dynamic models which operate with binary values. Gene states are modeled by a Boolean value, “on” or “off” (1 or 0). The global state of BN at every time-point is a vector of states of all genes and the state transition is defined by the vector of Boolean functions. The sequence of global states through time in dynamic models is termed as the trajectory. While there is only one trajectory for deterministic BN, the stochastic PBN provides multiple possible trajectories which represent different realizations of BN.

Bayesian networks are another class of methods [13], which represent relations between genes based on directed acyclic graphs (DAGs) and their conditional probabilities. Bayesian networks can be operated with continuous and discrete variables on continuous and discrete time. The extension of Bayesian networks is dynamic Bayesian networks (DBNs) [14], which introduce time delay between genes.

As an alternative to highly detailed and complex BN, PBN and DBN, the information theoretic models [15] emerged to provide the simple approach to perform analysis on the whole genome scale. According to the selected measures of relevance, there are two major categories based on correlation or mutual information (MI). One typical example of the first category is the weighted network analysis (WGCNA) [16]. Representative methods in the second category include the relevance networks (RN) [17], the maximum relevance/minimum redundancy network (MRNET) [18], the context likelihood relatedness (CLR) [19] and the algorithm for the reconstruction of accurate cellular networks (ARACNE) [20].

Methods which are able to establish causality are mostly graph based. For example, the method of GeneNet [21] converts correlation network into partial correlation graphs and further establishes partial ordering of nodes based on the covariance matrix. The method of GENIE3 [22] solves a regression problem for every gene using tree-based ensemble methods. The method of generalized local learning (GLL) performs local learning and feature selection in graphs [23, 24].

Based on survey of existing methods, we highlight six characteristics of a GRN inference approach which ideally should be considered in the state-of-the-art method: accuracy, ability to capture dynamics of temporal data, differentiation of direct and indirect regulations, detection of directionality of the link, assigning the most informative function to the link, and computation efficiently on large datasets.

Boolean Function Network (BFN) is a two-step GRN reverse-engineering approach to achieve the above six aims. At the first step, BFN identifies pairwise dependencies to explore directionality, optimal Boolean functions and time delays. At the second step, it tests directness of relations established in the first step. The purpose of ensuring of directness of the gene regulatory relations is to achieve the clear structural representation of GRN and reduce the number of false positive links. The output of algorithm is controlled by two threshold parameters *p*_1_ and *p*_2_ which set the significance level of each test in the first and the second step.

As comparison of performance, we evaluate the accuracy of BFN and Boolean Network [25], Dynamic Bayesian Network [26], information theory based and graph based GRNI methods [27]. The prior knowledge in form of list of regulators can be incorporated to BFN as well in order to enhance accuracy of prediction.

BFN can be considered as dynamic GRNI since it uses adaptive time delay between genes. Another important characteristic of BFN which is seldom addressed by the most existing approaches is the ability to identify the regulatory function describing the relation between gene pairs in GRNs. Among the methods reported in literature, the Boolean models (BN and PBN) are few methods that have such capacity. However, these methods use a limit number of Boolean functions, such as three Boolean gates (AND, OR, NOT) and their combinations. We will extend the number of Boolean functions in the proposed approach to describe the complex nature of regulatory relations.

Furthermore, BFN has low computational complexity and computation time. Therefore, it can be applied to high-throughput data, which is demonstrated for whole genome *S*. *cerevisiae* dataset in the section of “Results”.

## Results

### Method overview

The proposed BFN method is based on a hidden Markov model (HMM). We assume that the true status of a gene at discrete moment of time is a hidden state, while the measured expression level of gene transcript at the specific time is an observation. In a nutshell, the BFN method consists of two major steps: identification of pairwise dependencies between genes by Test 1 and the subsequent check whether those links are direct or indirect by Test 2.

**Fig 1:**
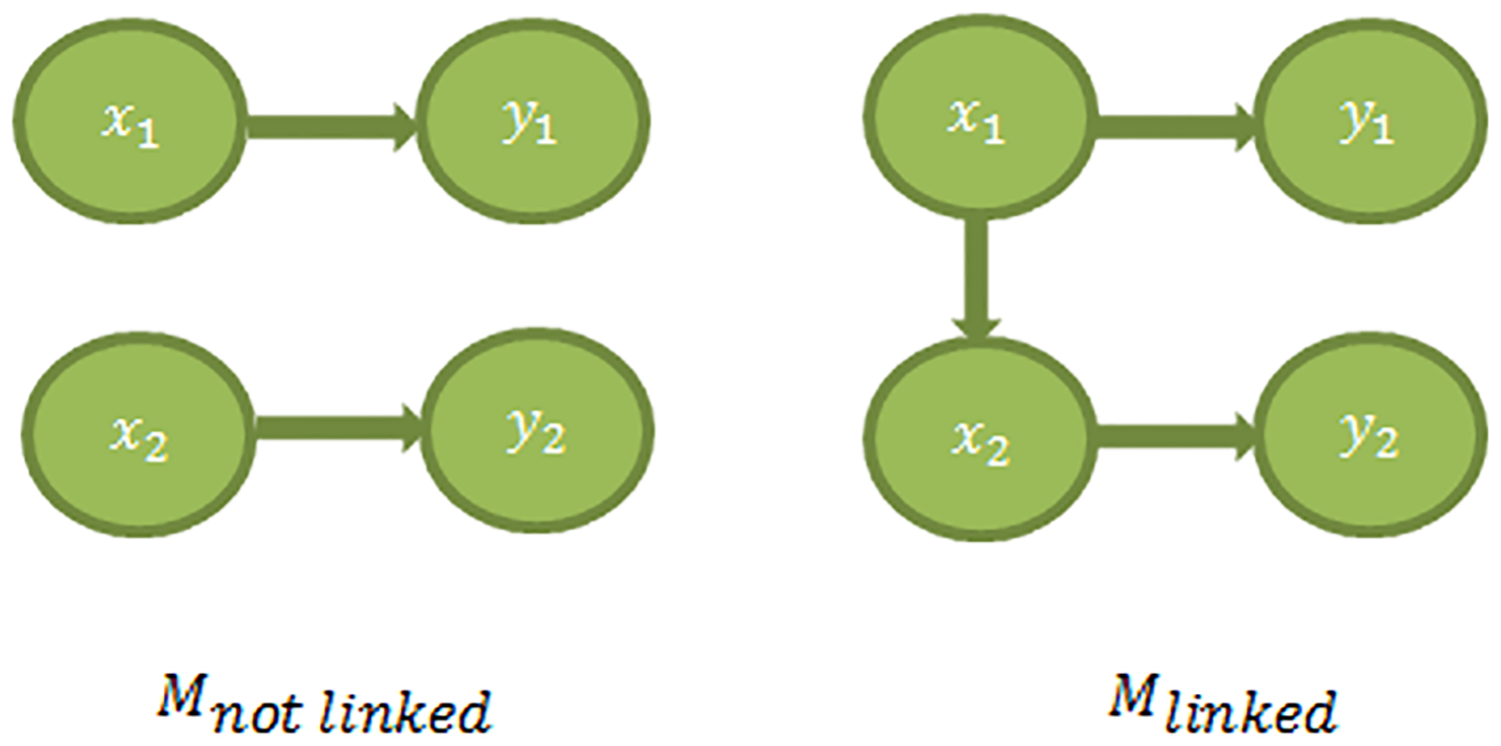
Linked model vs not-linked model. Model M _not linked_ assumes that Gene 1 and Gene 2 are unrelated; whereas Model M _linked_ assumes that Gene 1 regulates Gene 2. For both models, *x*_1_ and *x*_2_ are hidden gene states; while *y*_1_ and *y*_2_ are observed values.

**Fig 2:**
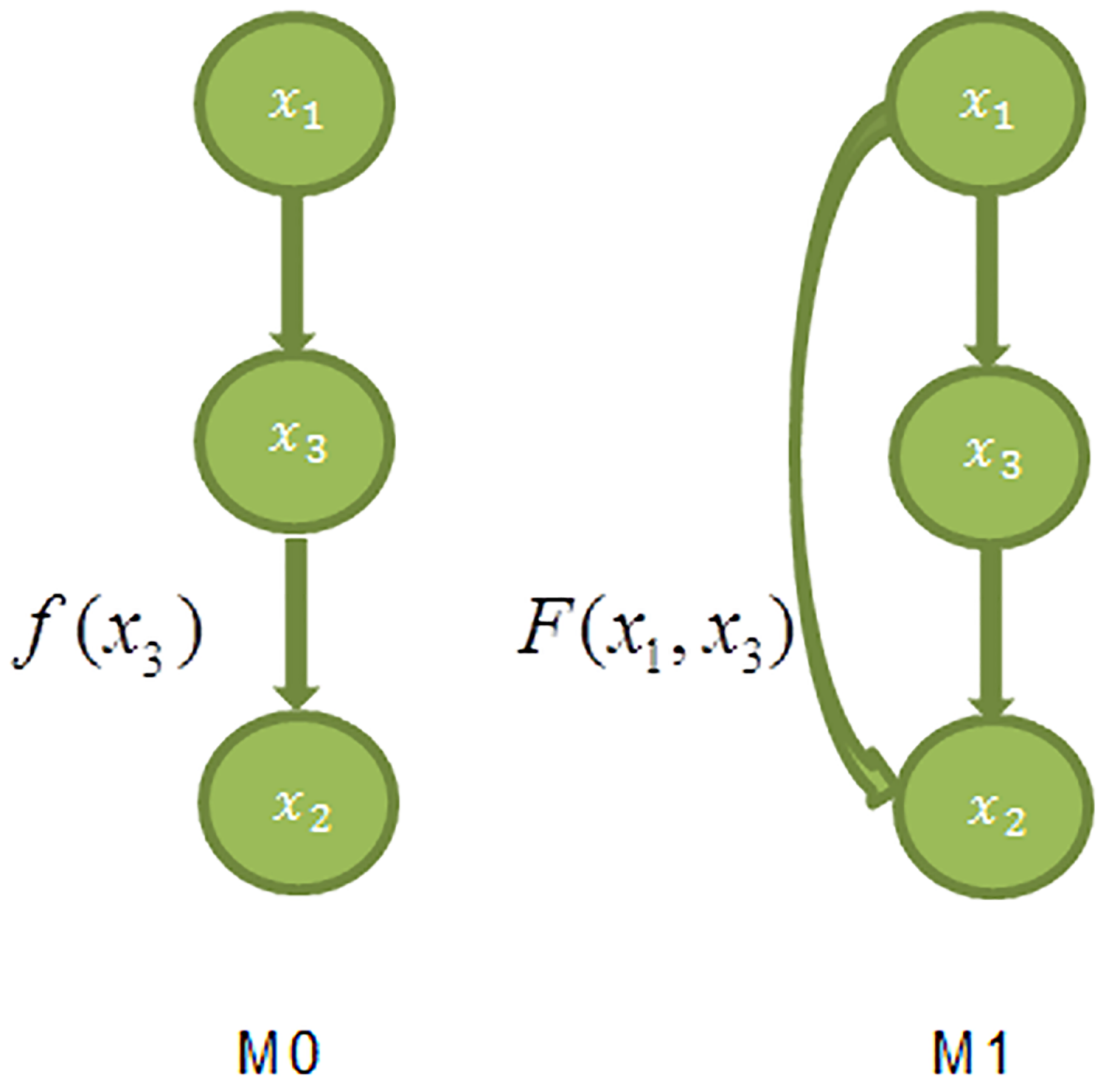
Indirect model M0 vs direct model M1. In the indirect model M0, gene *x*_1_ regulates gene *x*_2_ through intermediate gene *x*_3_; while in the direct model M1, both *x*_1_ and *x*_3_ regulate gene *x*_2_ directly. In this figure for the sake of simplicity we omit depicting observed values *y*_*i*_.

Both Test1 and Test 2 are based on the comparison of likelihoods of two alternative models. Test 1 search for the best Boolean function and time delay between two genes, which would maximize the likelihood ratio of a model with a link over a model without a link. Fig 1 provides a graphical illustration of Test 1. Analogously, Test 2 is illustrated with Fig 2 that compares the likelihoods of the model with a direct link and that with an indirect link. The reason why Test 2 is needed is that it helps to avoid collisions in causation order upon structural network reconstruction. Suppose, we have detected the following links *x*_1_ → *x*_2_, *x*_1_ → *x*_3_, *x*_2_ → *x*_3_ by Test 1. As it is shown at Fig 2 there are two ways to place those links at the map. In the first scenario, *x*_1_ has indirect effect on *x*_3_ through *x*_2_. In the second scenario, both *x*_1_ and *x*_2_ regulate *x*_3_ directly. Therefore the question arises which one of the two maps is correct? With the increasing number of genes, the resulting network can differ dramatically if the directness of the links is not tested. Further method’s details are explained in the section of Materials and Methods.

The proposed BFN method needs time course data to discover the regulatory relationships. The minimum number of time points required for proper inference is 10. To investigate the performance of BFN, we will use the widely used data of yeast expression in Spellman et al. [28]. Details about the dataset and its preprocessing are described in the section of Materials and Methods.

### Whole genome analysis and comparison to the other methods

Ma et al. [27] provided the combined binding and knockout data in 3 golden-standard networks for various levels of significance from the most conservative gene relationship #1 to the most liberal gene relationship #3. They used those as reference to evaluate the performance of 18 statistical approaches over 4 data types based on the assessments of 3 combined statistical metrics. We evaluate the performance of the proposed BFN with those 18 methods by the same assessment metrics. However, those data sets tested in Ma et al. [27] do not contain sufficient time course data for the application of the proposed BFN method. Thus, we will use the dataset in Spellman et al. [28] that is of the same data type for observational data obtained across time and/or environmental conditions studied in Ma et al. [27].

For BFN, we ran Test 1 and Test 2 consecutively with the p-value threshold of 0.05 for both tests. The time delay range is set to be the range from 0 to 5 on the Spellman dataset. 185 genes are assigned as TFs in according to SGD (Saccharomyces Genome Database) [29], which are served as sources. The list of genes and TFs can be found in the columns 1 and 2 of S7 Table. We found the gene relationships for the entire network that are comprised of 335531 direct links in S1 Table. Table 1 summarizes the performance of the BFN method for 3 networks and the details are explained in S2 Table. The performance in Table 1 is assessed with seven metrics: sensitivity or true positive rate (TPR), specificity or true negative rate (TNR), precision or positive predictive value (PPV), negative predictive value (NPV) and 3 combined metrics which show Euclidean distance from the ideal performance point ($C1=\sqrt{{\left(1-sensitivity\right)}^{2}+{\left(1-specificity\right)}^{2}}$, $C2=\sqrt{{\left(1-PPV\right)}^{2}+{\left(1-NPV\right)}^{2}}$, $C3=\sqrt{{\left(1-sensitivity\right)}^{2}+{\left(1-PPV\right)}^{2}}$). Since these three combined metrics describe the distance from the ideal performance point (e.g., *sensitivity* = 1, *specificity* = 1 in C1), the smaller score indicates the better performance.

**Table 1:** Performance of BFN on the whole genome Spellman dataset measured with seven accuracy metrics for three gold-standard regulatory relation datasets.

Reference	TPR	TNR	PPV	NPV	C1	C2	C3
GRN#1	0.3482	0.7548	0.0287	0.9823	0.6964	0.9714	1.1697
GRN#2	0.3276	0.7585	0.0286	0.9811	0.7144	0.9716	1.1814
GRN#3	0.3188	0.7561	0.0283	0.9803	0.7235	0.9719	1.1867

According to the scores reported in Table 1, the performance of the BFN method is similar for three gold-standards. The performance is slightly better for the gold-standard GRN #1 which contains only regulatory relations with highly significant links.

Table 2 provides an overview of BFN performance compared to the performance of 18 statistical approaches reported in Ma et al. [27] by 3 combined metrics. Each sub table in Table 2 reports the performance comparison to every specific combined metric, C1, C2 or C3. The performance comparison incorporates the followings. 1) The average score over 18 approaches and 4 observational datasets obtained by changing time and/or environmental conditions (i.e., Gresham et al. [30], Gasch et al. [31], Smith et al.[32], Yeung et al.[33]) measured with one of three GRN gold-standard networks. 2) The corresponding BFN score. 3) The best values among 18 methods in each of 4 datasets.

**Table 2:** Performance comparisons of BFN with 18 statistical approaches evaluated by Ma et al. [27]. Each sub table represents the comparison by the score of C1, C2 or C3 metric correspondingly.

Golden standard network used as reference	Average of C1 scores over 18 approaches and 4 observational datasets	C1 score of network obtained with BFN	The best values of C1 among 18 methods in each of 4 datasets.			
			Gresham	Gasch	Smith	Yeung
GRN#1	0.86	0.70	0.70	0.69	0.72	0.77
GRN#2	0.87	0.71	0.70	0.69	0.73	0.79
GRN#3	0.87	0.72	0.70	0.71	0.73	0.80
Golden standard network used as reference	Average of C2 scores over 18 approaches and 4 observational datasets	C2 score of network obtained with BFN	The best values of C2 among 18 methods in each of 4 datasets.			
			Gresham	Gasch	Smith	Yeung
GRN#1	0.97	0.97	0.97	0.95	0.93	0.95
GRN#2	0.97	0.97	0.97	0.96	0.94	0.95
GRN#3	0.97	0.97	0.97	0.97	0.94	0.95
Golden standard network used as reference	Average of C3 scores over 18 approaches and 4 observational datasets	C3 score of network obtained with BFN	The best values of C3 among 18 methods in each of 4 datasets.			
			Gresham	Gasch	Smith	Yeung
GRN#1	1.23	1.17	1.04	1.07	1.00	1.00
GRN#2	1.23	1.18	1.04	1.07	1.00	0.99
GRN#3	1.24	1.19	1.05	1.07	1.00	1.00

From these comparisons, the performance of BFN is better than the average results. The performance in C1 and C3 metrics shows that the BFN can achieve improvements on the average. In particular, the improvement of BFN is appealing for C1 metric (sensitivity and specificity) across all GRNs gold-standards. When compared with the best individual scores, the BFN has better performance in C1 metric than any method applied to Smith and Yeung datasets, while no advantage can be seen for C2 and C3 metrics. It should be noted that performance ranks of those methods in Table 2 differ with distinct metrics. For example, the method producing the best score (0.69) for C1 metric (sensitivity and specificity) will generate the worst score (0.98) in C2 (PPV and NPV) in the Gasch dataset when GRN #1 is used as the gold-standard. Thus it is rather difficult to make comparison of performance among individual approaches and average scores seem to be more informative.

### Performance comparison with Boolean network and Bayesian network models on simulated data

**Fig 3:**
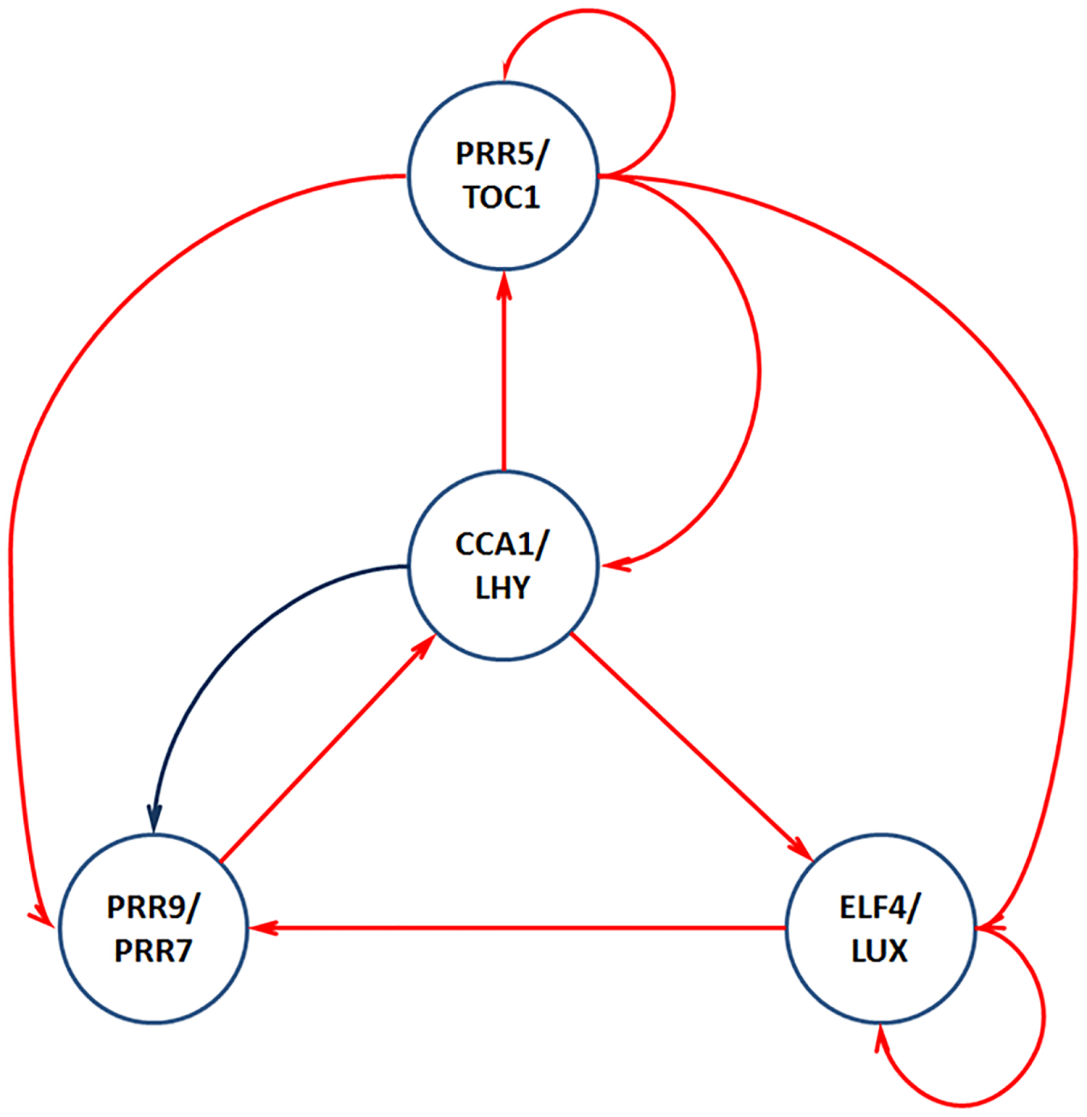
Circadian clock model. Red links represent negative regulation and blue link is a positive regulation.

De Caluwé et al. [34] proposed a compact model of circadian clock in *Arabidopsis thaliana* which consists of 8 genes paired (based on expression pattern similarity) into 4 modules (Fig 3).

**Fig 4:**
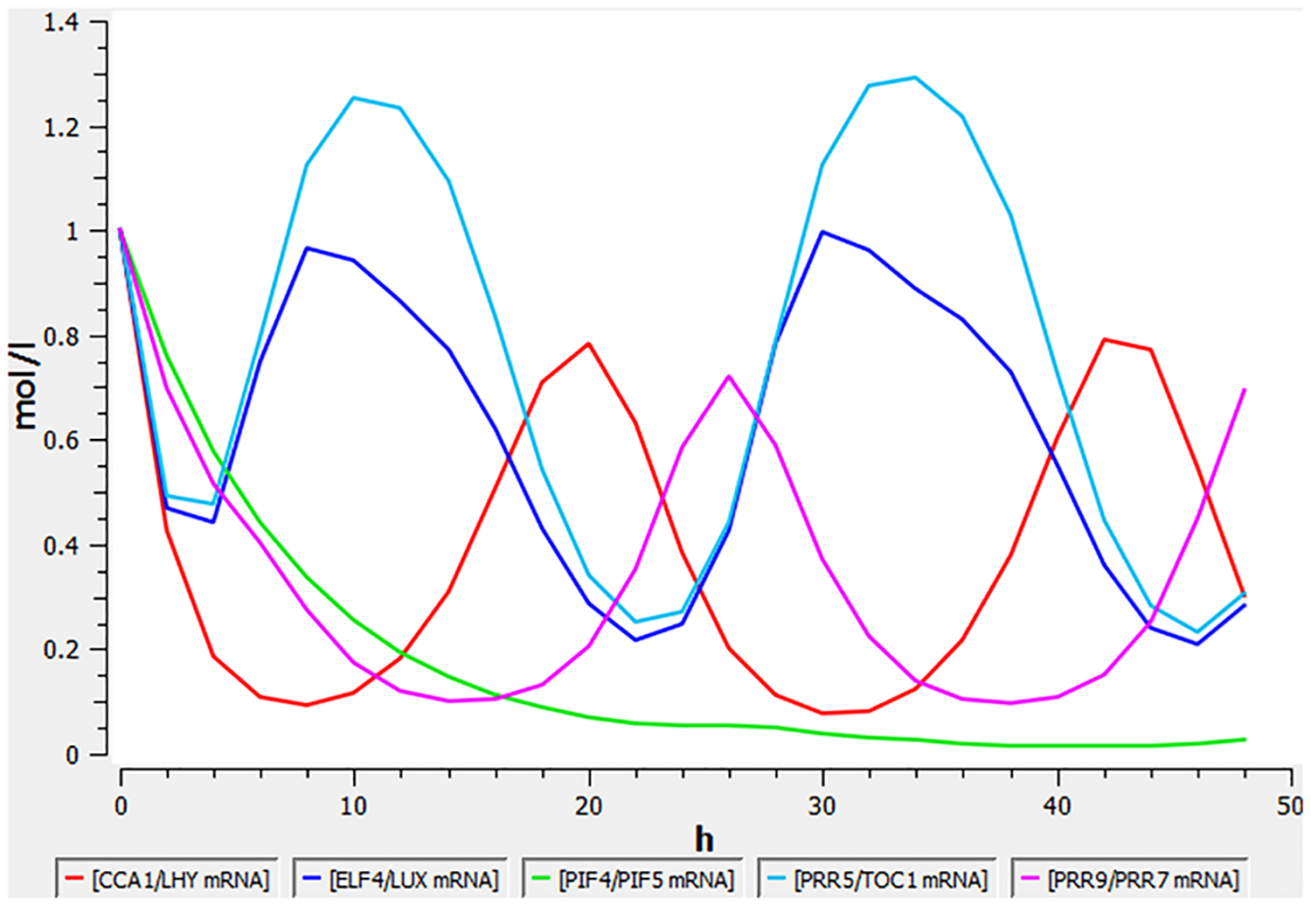
Change of mRNA concentration over time for CCA1/LHY, ELF4/LUX, PIF4/PIF5, PRR5/TOC1, PRR9/PRR7 gene pairs.

We run the corresponding computational model obtained from BioModels Database [35] (BIOMD0000000631) with simulator for biochemical networks COPASI (ver. 4.19) [36] to acquire the time course transcriptome data. Fig 4 depicts COPASI generated time course data representing the concentration of 5 gene-pairs transcripts over 50 hours period with intervals of 2 hours and the initial state equals 1.

We compared the ability of BFN to reconstruct gene regulations shown in Fig 3 with two Boolean Network methods of REVEAL [37] and Best-Fit [38] (included in the R “BoolNet” package [25]) and the Dynamic Bayesian Network (DBN) inference (coded in the R “G1DBN” R package [26]). The REVEAL approach has failed to infer any network from given data, while the best results of Best-Fit, G1DBN and BFN are provided in Table 3.

**Table 3:** Inference results of Boolean network (Best-Fit with optimal discretization k-means and maxK = 2), Dynamic Bayesian Network (with least squares estimation, and default parameters alpha1 = 0.5 and alpha2 = 0.05 for first order dependencies and full dependencies correspondingly) and Boolean Function Network (maxTimeDelay = 10 and p1 = 0.005).

Best-Fit	G1DBN	BFN
CCA1/LHY = <f(CCA1/LHY,PRR9/PRR7){0010}>	(PIF4/PIF5; PIF4/PIF5)	(CCA1/LHY; PRR9/PRR7; f = 1, t = 3)
PRR9/PRR7 = <f(PRR9/PRR7){01}>	(CCA1/LHY; ELF4/LUX)	(CCA1/LHY; PRR5/TOC1; f = 2, t = 1)
PRR5/TOC1 = <f(CCA1/LHY,ELF4/LUX){0100}>	(CCA1/LHY; PRR5/TOC1)	(CCA1/LHY; ELF4/LUX; f = 2, t = 1)
PRR5/TOC1 = <f(CCA1/LHY,PRR5/TOC1){0100}>	(ELF4/LUX; PRR5/TOC1)	(PRR9/PRR7; CCA1/LHY; f = 2, t = 2)
ELF4/LUX = <f(CCA1/LHY,ELF4/LUX){0100}>	(CCA1/LHY; CCA1/LHY)	(PRR9/PRR7; PRR5/TOC1; f = 1, t = 4)
ELF4/LUX = <f(CCA1/LHY,PRR5/TOC1){0100}>	(ELF4/LUX; ELF4/LUX)	(PRR9/PRR7; ELF4/LUX; f = 1, t = 4)
PIF4/PIF5 = <f(PRR9/PRR7,PIF4/PIF5){0001}>	(PRR9/PRR7; PRR9/PRR7)	(PRR5/TOC1; CCA1/LHY; f = 1, t = 4)
	(PRR5/TOC1; ELF4/LUX)	(PRR5/TOC1; PRR9/PRR7; f = 2, t = 2)
	(PRR9/PRR7; CCA1/LHY)	(PRR5/TOC1; ELF4/LUX; f = 2, t = 5)
	(PRR9/PRR7; PIF4/PIF5)	(ELF4/LUX; PRR9/PRR7; f = 2, t = 2)
	(ELF4/LUX; PIF4/PIF5)	(ELF4/LUX; PRR5/TOC1, f = 1, t = 1)
	(ELF4/LUX; PRR9/PRR7)	

Table 4 contains the performance analysis for Boolean Network (Best-Fit), Dynamic Bayesian Network (G1DBN) and Boolean Function Network (BFN). To make a comprehensive comparison, we conduct the Boolean Network method of Best-Fit with three different discretization approaches (k-means, edgeDetector and scanStatistic). The result with edgeDetector is removed from Table 4 due to the poor performance. We evaluate the performance of k-means and scanStatistic by three options for maximum arity of Boolean functions (2, 3 or 4). The DBN was tested with different approaches to regression estimates (Huber, Tukey and Least Squares). However, only the Least Squares method managed to converge in given number iterations.

**Table 4:** Performance characteristics for Boolean Network, Dynamic Bayesian Network and Boolean Function Network.

Method	TPR(sensitivity)	TNR(specificity)	PPV(precision)	NPV
Best-Fit (k-means, maxK = 2)	0.50	0.69	0.56	0.64
Best-Fit (scanStatistic, maxK = 2)	0.30	0.77	0.50	0.59
Best-Fit (k-means, maxK = 3)	0.50	0.50	0.42	0.58
Best-Fit (scanStatistic, maxK = 3)	0.10	0.38	0.11	0.36
Best-Fit (k-means, maxK = 4)	0.50	0.50	0.42	0.58
Best-Fit (scanStatistic, maxK = 4)	0.10	0.38	0.11	0.36
DBN (Least Squares)	0.60	0.60	0.50	0.69
BFN	0.70	0.73	0.64	0.79

As it can be seen from Table 4, the BFN performs better in this example with respect to most performance measures (sensitivity, specificity, precision and NPV). Moreover, the BFN can assign both Boolean function and time delay for each relation.

### Performance evaluation of BFN by Saccharomyces Genome Database (SGD) regulatory information

**Fig 5:**
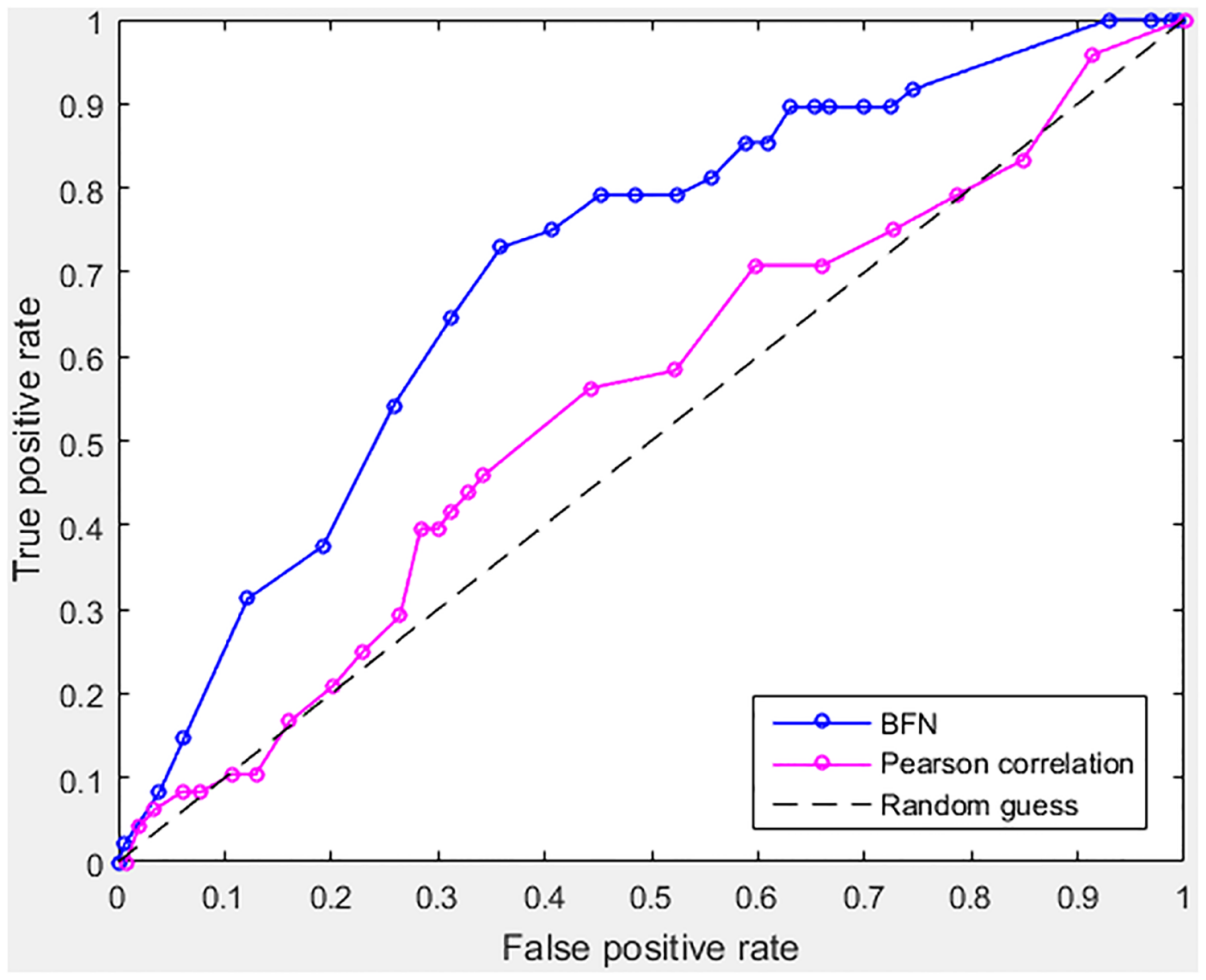
ROC curves of BFN (based on Test1) and Pearson correlation applied to the set of 103 cell-cycle regulated genes. The BFN curve is depicted in blue and the Pearson correlation curve is shown in magenta.

The performance improvement of BFN over the classical approach of Pearson correlation is evaluated by the Saccharomyces Genome Database (SGD) regulatory information. We focus on the 103 cell-cycle regulated *S*. *cerevisiae* genes annotated from previous studies [28]. Among them, 7 are TFs according to SGD and they are used as source genes. The relations discovered with each method were assessed with the SGD regulatory information. The detailed information about numbers of true positive, false positive, true negative and false negative links obtained with BFN at varying p-value thresholds of Test1 is described in S3 Table. Moreover, S3 Table contains the list of all possible links between cell-cycle regulated genes (listed in columns 3 and 4 of S7 Table) with corresponding p-values. Two ROC curves describing the performances of BFN and Pearson correlation are shown in Fig 5. With significant improvement in sensitivity and specificity, the proposed BFN method outperforms the classical approach of Pearson correlation.

### Cell cycle genes and their succession along cell cycle phases

Conventionally cell cycle is partitioned into four consecutive phases: mitosis (M), gap1 (G1), synthesis (S) and gap 2 (G2). In this study, we focus on the list of 103 cell-cycle genes [28] along with their corresponding cell phase labels assigned according to literature (Columns 5 and 6 in S7 Table). We allow all these 103 genes to be sources and targets. The cut-off values of *p*_1_ = 0.005 and *p*_2_ = 0.05 are used for Test1 and Test2 correspondingly. The time delay range varies from 1 to 5 sample time points. We consider links of positive Boolean functions only. As a result, we obtained 68 links in S4 Table. We check the consistency of 68 relations discovered via the BFN method with cell phase annotation of these 103 genes. If the label of one target gene is the same as its source’s label or is successive to the source’s label, then this relation is consistent with cell cycle information. It turns out that all these 68 links listed in S4 Table are consistent with cell cycle information.

**Fig 6:**
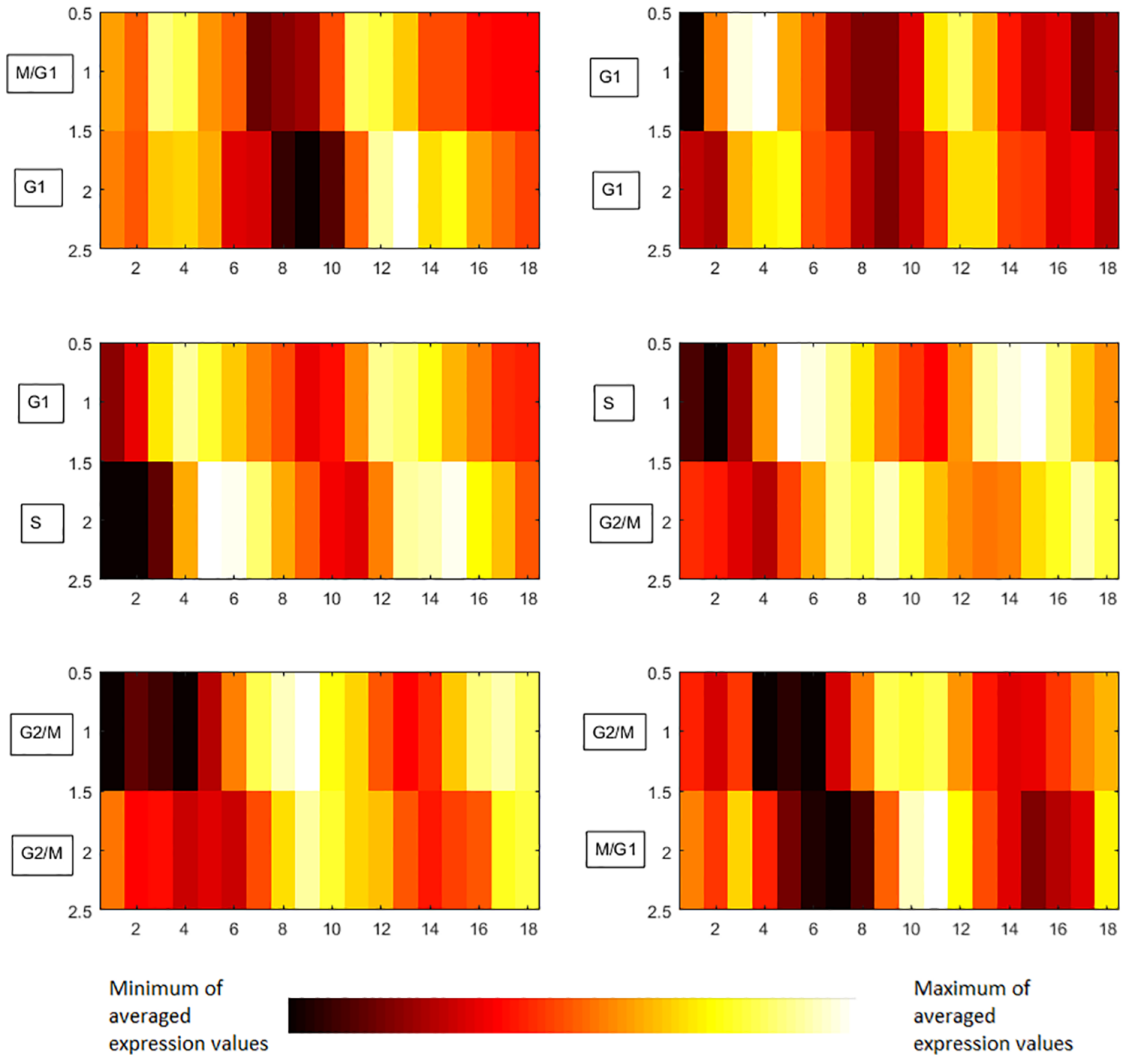
Six largest groups of positive links discovered by BFN on the list of 103 genes annotated with cell cycle phases. On the horizontal axis, there are time points. On the vertical axis, there are the average expression values of source (up) and target (bottom) genes which belong to corresponding group of links.

Fig 6 shows the average expression values for source and target genes in 6 largest groups of positive links representing consecutive phases of cell cycle identified with BFN method for the set of 103 genes. Most of the links in Fig 6 have the time delay parameter equals to 1, except the links of S->G2/M where the time delay parameter is identified as 2. Thus, the relationship and the time delay parameters discovered by BFN can reveal the relationship of these 103 genes hidden in transcriptome data correctly.

### Functional enrichment analysis

The capacity of BFN to identify a Boolean function and a time delay for each regulatory relation can be utilized not only for the reconstruction of gene regulatory map but also the analysis of gene sets. For each transcription factor (TF), the pool of target genes can be naturally divided into groups according to their Boolean functions and time delay parameters detected by BFN. For each TF, the results of the whole genome *S*. *cerevisiae* analysis (S1 Table) can be divided up to 36 groups because there are 6 possible Boolean functions and 6 possible time delays. S5 Table contains the results of gene ontology (GO) and pathway enrichment for detected groups of target genes (with TFs excluded from target groups) which are positively regulated by each of 185 TFs with time delays of 0 and 1. Annotations were obtained with the YeastMine, which is the embedded tool in SGD [29] for data searching, retrieving and annotating. In total, there are 210 out of 370 possible gene groups received annotation of Molecular Function, Biological Process or Pathway.

The obtained information is useful both for elaboration of TF’s function and for establishing candidate genes of regulatory targets. To illustrate the former case, we consider the YHL020C (OPI1) transcription factor. The annotation by SGD shows that the regulatory targets are related to the “carboxylic acid biosynthetic process” (GO: 0046394). When we consider the subgroup according to time delay 0 (No. 37 in S5 Table), the targets of YHL020C suggest the “methionine biosynthetic process” (GO:0009086) annotation which is one more specific GO category positioned two levels below in GO hierarchy. Therefore, the BFN suggests that YHL020C can be involved in the positive regulation of biosynthesis of methionine and this process is time-constrained within one time interval.

As an example for the second type of information that can be extracted from the functional annotation results by BFN, we consider the YPR008W (HAA1) transcription factor (group No. 208 in S5 Table). According to the results positively regulates genes, the BFN suggests that the target genes are YML100W (TSL1), YBR126C (TPS1), YDR074W (TPS2) and YMR261 (TPS3) with time delay 1. Protein subunits TPS1, TPS2, TSL1 and TPS3 constitute the alpha,alpha-trehalose-phosphate synthase complex which converts glucose 6-phosphate plus UDP-glucose to trehalose in two-steps trehalose biosynthetic pathway. Currently, only TSL1 is a regulatory target of HAA1 registered in SGD. However, according to our functional annotation, the other three genes YBR126C(TPS1), YDR074W(TPS2), YMR261(TPS3) are also strong candidates to be the regulatory targets of YPR008W(HAA1) together with YML100W(TSL1) because they show highly significant (p-value = 0.0001) pathway enrichment. The detailed information about the list of genes referring to each GO category is not included in S5 Table for table concreteness. But they can be easily obtained if the list of target genes (excluding TFs) for a specific TF in S1 Table is loaded into YeastMine.

### Transcriptional regulation of metabolic pathway

**Fig 7:**
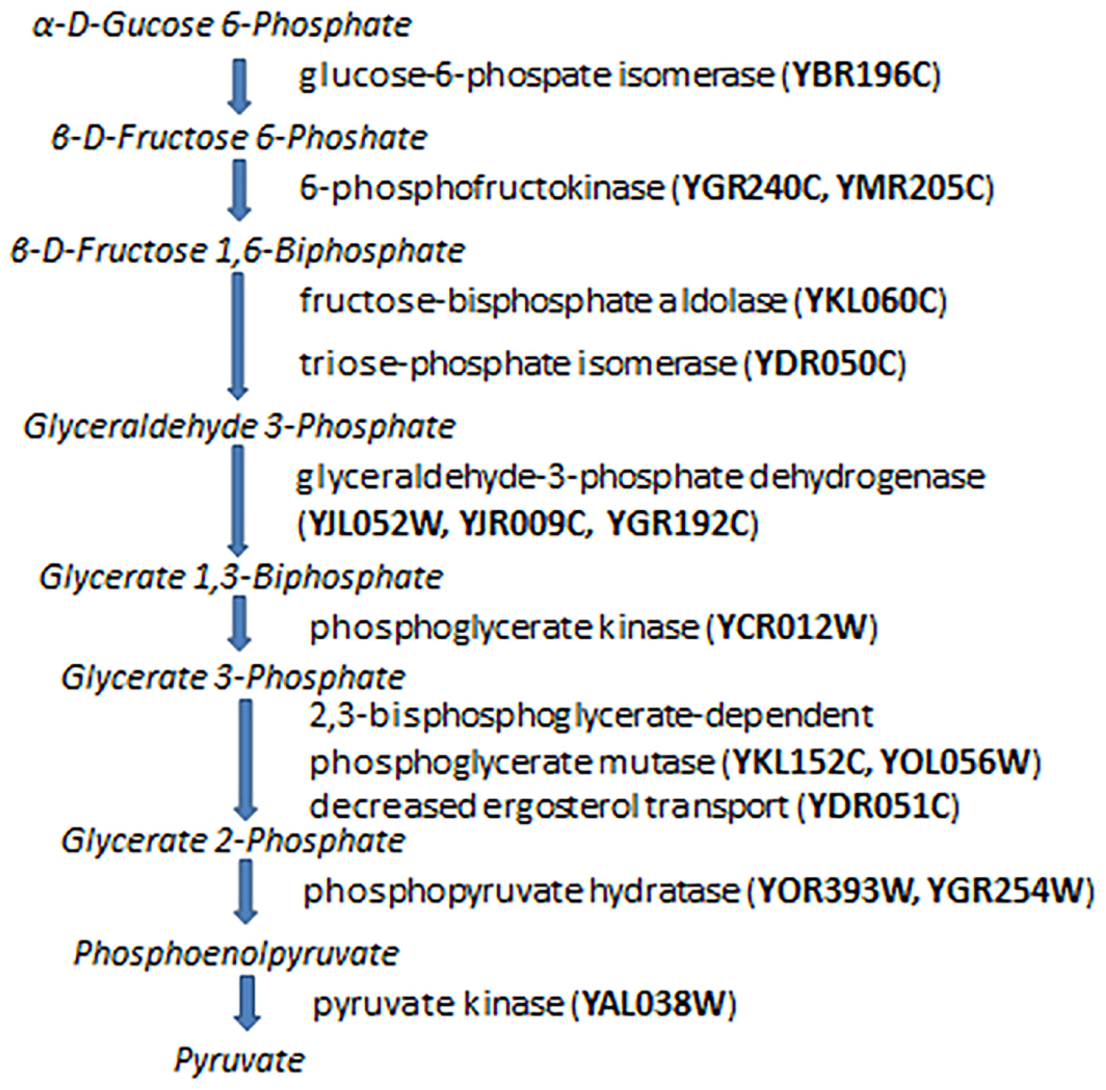
Glycolysis metabolic pathway in *S*. *cerevisiae*. Intermediates of glycolysis are expressed in italic. The names of enzymes whose abundance controls conversion of one intermediate into another are on the right side of arrow.

**Fig 8:**
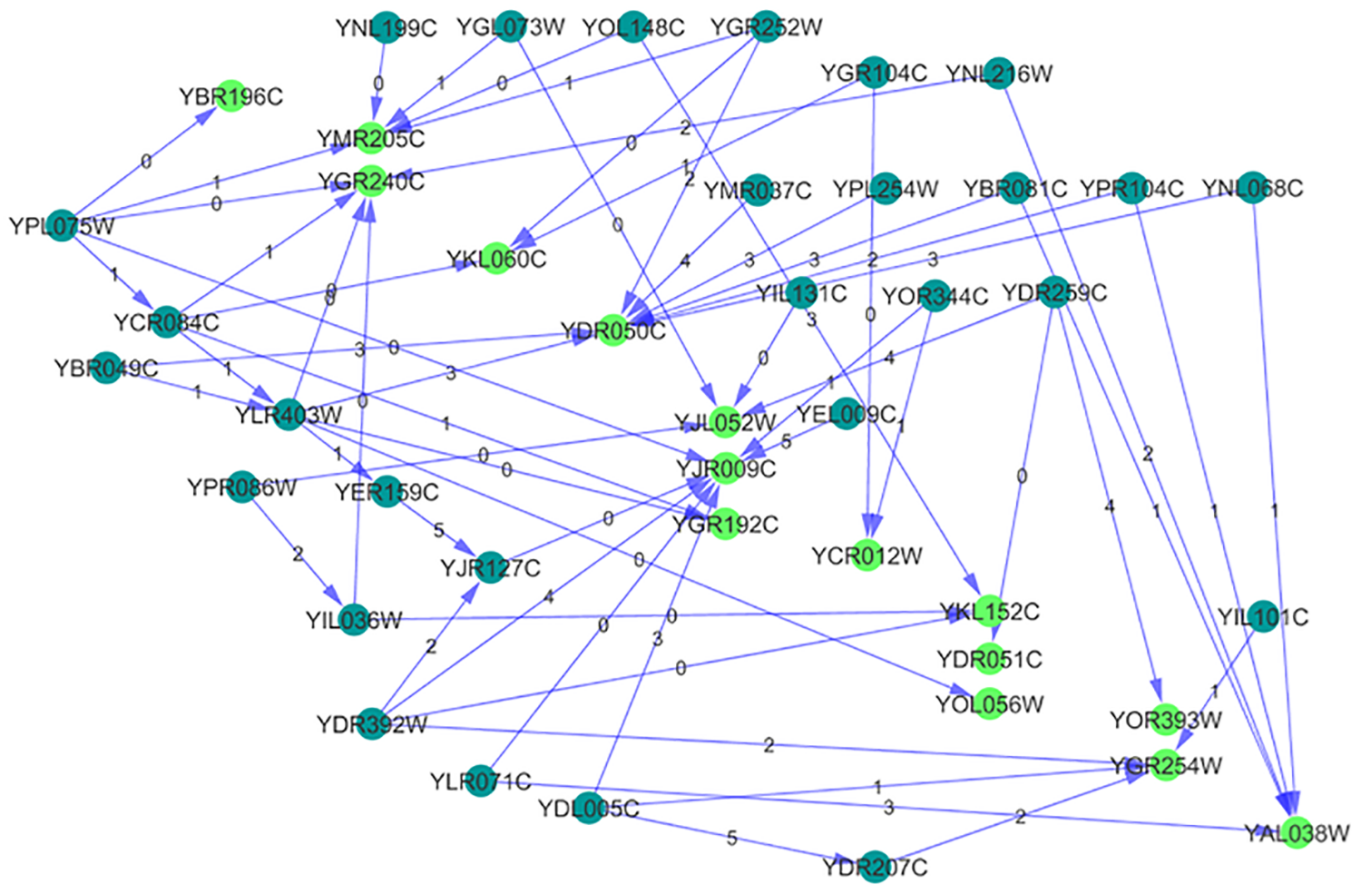
Part of the reverse-engineered GRN (with true positive links only). Map displays the regulation of 15 genes encoding 9 enzymes involved in glycolysis with transcription factors. Genes are colored with light green and TFs regulating them are colored with dark green. All links are positive and labeled with time delays.

Based on the references [39, 40], we schematically illustrate the conversion of D-glucose to Pyruvate in the process of glycolysis in Fig 7. Using the SGD regulatory information for genes encoding 9 essential glycolytic enzymes (provided in Fig 7) as the reference to eliminate false positive results, we are able to highlight the regulatory subnetwork for glycolytic genes from our whole-network results in S1 Table. The results can be found in S6 Table which contains 147 regulatory relations out of 398 registered with SGD for these 9 genes. We use Cytoscape [41] to illustrate the simplified map with positive links only in Fig 8. In the map of Fig 8, there are two TFs that are well-known activators of glycolysis in yeast, GCR1 (YPL075W) and GCR2 (YNL199C). Remarkably in the map of Fig 8, YPL075C causes not only direct activation of genes but also induce cascade of TFs (YCR084C, YLR403W, YER159C, YJR127C) which also activate glycolytic genes.

## Discussion

The proposed BFN approach is a fast and efficient way to explore the regulatory relations between genes for further experimental analysis. By adjusting those two threshold parameters *p*_1_ and *p*_2_ which set significance level in two consecutive tests, we can trade off the numbers of false positive and false negative links. Thus, the smaller values of *p*_1_ and *p*_2_ are set, the more stringent restrictions we apply for the output. Consequently, the smaller size of output links will be generated with the smaller number of false positive links and the larger number of falsely rejected links. The user can utilize the prior knowledge about known regulatory relations to decide the level of significance. Moreover, the choice of *p*_1_ and *p*_2_ also depends on the number of time-points in dataset. The more time-points there are at disposal, the more links will be discovered to be significant, thus more stringent limits on *p*_1_ and *p*_2_ need to be applied. Even though the range of time delay is another parameter to be set, it can be easily decided based on the number of time points and biological knowledge such as periodicity of cell cycle or circadian rhythm as demonstrated in this study. The lower bound of time delay range should be chosen depending on whether we need to consider co-regulated sets of genes (time delay equals to 0) or we are only interested in pairwise relations when regulatory effect can be seen over time (time delay equals to 1 or higher). Based on our empirical experience, the maximum time delay should be no larger than one third of number of time-points in dataset and at least a half of the time interval between cell’s steady states.

Similar to any other computational methods based solely on transcriptome data, the BFN is not sufficient to reconstruct GRN entirely because the posttranslational modification should be taken in to account. Moreover, the observational dataset can reflect the status of GRN only under specific experimental conditions. However, the proposed BFN method can become valuable tool for biologists to reduce the search space for relations between genes. And it will help to recover the overall picture of regulatory pathways when it is applied to several related time-series data under different experimental conditions.

When the prior knowledge is available, such as a list of TFs, it can be integrated to the proposed BFN to reduce computational complexity and improve prediction precision. The apparent advantage of the BFN method is that it not only determines direct relationships between genes but also provides direction and Boolean function with time delay. The follow-up division into groups based on the assignment of Boolean functions and time delays to each relation can be incorporated to clustering and for the analysis of functional enrichment.

The proposed BFN can identify directness of a link between a pair of genes. It could be expanded to discover structures of three and more genes with higher computational complexity in future studies.

## Materials and methods

### *S*. *cerevisiae* dataset and its preprocessing

Spellman et al. [28] provide data from 3 microarray experiments with different synchronization techniques. For this study, we use data obtained with α-factor cells arrest since this experiment has the highest time resolution (the measurements of RNA were taken every 7 min). The total number of genes in data set is 6075 measured along 18 time-points. There are 59 genes excluded from analysis since there were 4 or more missing values for each gene. For the rest of genes, the missing values were replaced with spline extrapolation. Therefore the whole genome dataset analyzed in this study consists of 6016 genes.

### Discretization

The expression profile is the measured abundance of mRNAs for each determined point in time. The source for this type of data can be microarray, next generation sequencing and other types of biochip experiments. Hereafter, we arrange the variables (genes) horizontally and n is the number of genes. The observations (time-points) are arranged vertically with the total number of columns is m, n≫m. Naturally, the range of values varies greatly from one gene to another. In order to enable comparison of the expression profiles of different genes, the expression values have to be standardized to the same scale, i.e. converted to the standard range of [0, 1] for every gene. We will apply the approach of empirical cumulative distribution function (ECDF) transformation, which can be described as follows.

For each gene *x*_*i*_:

- Sort observations *x*_*ij*_ along *m* time-points in ascending order.
- Assign to corresponding observation a probability $p_{iI_{j}}=\frac{j}{m}$, *j* = 1…*m*, where *I* is array of sorting indices.

If there are ties (the same values of observations) for some genes, then the above standardization procedure can generate skewness. Thus, we use the following modified procedure for standardization in this study.

For each gene *x*_*i*:_

- Sort observations *x*_*ij*_ along *m* time-points in ascending order.
- Identify unique values *u*_*ik*_, *k* = 1…*K*, *K* < *m*
- For each unique value *u*_*ik*_:
  - Count the number of ties *c*_*k*_ for given unique value
  - Compute and assign to corresponding $x_{iI_{j}}$,…, $x_{iI_{j+c_{k}-1}}$ probabilities $\{p_{iI_{j}}\ldots p_{iI_{j+c_{k}-1}}\}=\frac{C+c_{k}}{m}$, where *I* is array of sorting indices and $C=\sum_{k}c_{k-1}$.

After applying the above standardization, we obtain the corresponding empirical cdf value ${\hat{F}}_{i}(t)$ for every gene *i* at time point *t*.

### Boolean Network and Boolean Functions

We define the Boolean network as a set of vertices *V* = {*x*_1_…*x*_*n*_} representing genes together with the set of all unary and binary Boolean functions **f** = {*f*_1_…*f*_6_,*F*_1_…*F*_42_} which defines relations between nodes.

Boolean function is a mapping of the form *f*: *B*_*k*_ → *B*, where *B* = {0,1} is a Boolean domain and *k* is arity of the function. For every *k* there exist a finite set of non-trivial Boolean functions which can be represented in the form of truth table. In Tables 5 and 6, we enumerate all possible non-trivial Boolean unary and binary functions correspondingly:

**Table 5:** Truth table for unary functions f1-f6. x1 is input of function (source gene) and x2 is output of function (target gene).

x1	x2					
	f1	f2	f3	f4	f5	f6
0	0	1	0	1	{0,1}	{0,1}
1	1	0	{0,1}	{0,1}	0	1

**Table 6:** Truth table for binary functions F1-F42. x1 and x2 are input of the function and x3 is output.

x1	x2	x3													
		F1	F2	F3	F4	F5	F6	F7	F8	F9	F10	F11	F12	F13	F14
0	0	0	0	0	0	0	1	1	1	1	1	{0,1}	{0,1}	{0,1}	{0,1}
0	1	0	0	1	1	1	0	0	0	1	1	0	0	0	0
1	0	0	1	0	1	1	0	0	1	0	1	0	0	1	1
1	1	1	0	0	0	1	0	1	1	1	0	0	1	0	1
x1	x2	x3													
		F15	F16	F17	F18	F19	F20	F21	F22	F23	F24	F25	F26	F27	F28
0	0	{0,1}	{0,1}	{0,1}	{0,1}	0	0	0	0	1	1	1	1	0	0
0	1	1	1	1	1	{0,1}	{0,1}	{0,1}	{0,1}	{0,1}	{0,1}	{0,1}	{0,1}	0	0
1	0	0	0	1	1	0	0	1	1	0	0	1	1	{0,1}	{0,1}
1	1	0	1	0	1	0	1	0	1	0	1	0	1	0	1
x1	x2	x3													
		F29	F30	F31	F32	F33	F34	F35	F36	F37	F38	F39	F40	F41	F42
0	0	0	0	1	1	1	1	0	0	0	0	1	1	1	1
0	1	1	1	0	0	1	1	0	0	1	1	0	0	1	1
1	0	{0,1}	{0,1}	{0,1}	{0,1}	{0,1}	{0,1}	0	1	0	1	0	1	0	1
1	1	0	1	0	1	0	1	{0,1}	{0,1}	{0,1}	{0,1}	{0,1}	{0,1}	{0,1}	{0,1}

Besides all possible non-trivial Boolean functions with unique definite output, we also consider functions with two possible outputs, {0, 1}, which means that either 0 or 1 may appear in the output for the same input assignment. In Table 5, function f1 (equivalence) represents upregulation of gene x2 by gene x1. Function f2 (negation) stands for downregulation of gene x2 by gene x1. Functions f3 and f4 reflect relation of necessity and its negation correspondingly. That is, function f3 explains condition “gene x2 cannot be turned on unless gene x1 is turned on” and function f4 states opposite “gene x2 cannot be turned off unless gene x1 is turned on”. Functions f6 express sufficiency and f5 is its negation. If gene x1 is sufficient for gene x2 it means that knowing that gene x1 is on we can claim that gene x2 is on as well. However, it is not legit to assert that if gene x1 is off then gene x2 is off too. Whilst function f5 stands for statement “if gene x1 is on then gene x2 must be off”.

In Table 6, functions F1-F42 with binary inputs may or may not have simple and intuitive forms. For instance, F1 realizes an AND gate of two inputs; yet F24 does not have a simple Boolean functional form. In this study, we concern primarily pairwise dependencies in the gene network. The Boolean functions with binary inputs are auxiliary in determining the directness of links.

### Test 1

In order to identify the pairwise dependencies between genes, we examine two models for every possible pair of genes. One model represents the situation where genes are linked and the other model suggests there is no link between genes under consideration.

Assume that *y*_1_(*t*) and *y*_2_(*t*) are continuous observed values of Gene 1 and Gene 2 at time point *t* respectively. The notations of *x*_1_(*t*) and *x*_2_(*t*) are the corresponding discrete latent variables. The notation of *τ* represents the time delay between genes. For example, *τ* = 1 means the time delay of 7 minutes for the expression data of α -factor cells arrest in Spellman et al. [28]. Two competitive models are shown at Fig 1. In order to establish which one of the models explains data better, we use the likelihood ratio to evaluate:

$$R=\frac{L_{linked}}{L_{notlinked}}.$$

The larger this ratio is, the more significant the link is. The likelihoods of models can be written respectively:

$$L_{not\;linked}=\prod_{t}\sum_{\begin{array}{l} x_{1}(t)\\ x_{2}(t) \end{array}}P(x_{1}(t))\cdot P(x_{2}(t+\tau ))\cdot P(y_{1}(t)|x_{1}(t))\cdot P(y_{2}(t+\tau )|x_{2}(t+\tau )),$$ (1)

$$L_{linked}=\prod_{t}\sum_{\begin{array}{l} x_{1}(t)\\ x_{2}(t) \end{array}}P(x_{1}(t))\cdot P(x_{2}(t+\tau )|x_{1}(t))\cdot P(y_{1}(t)|x_{1}(t))\cdot P(y_{2}(t+\tau )|x_{2}(t+\tau )).$$ (2)

where the product is taken over all time points; at each time point *t* the likelihood score is marginalized over all possible latent variable states of *x*_1_(*t*) and *x*_2_(*t*).

According to Bayes’ theorem,

$$P(y_{k}(t)|x_{k}(t))=\frac{P(x_{k}(t)|y_{k}(t))P(y_{k}(t))}{P(x_{k}(t))}.$$ (3)

When *P*(*y*_1_(*t*)|*x*_1_(*t*)) and *P*(*y*_2_(*t*)|*x*_2_(*t*)) in formulas (1) and (2) are replaced with (3), we obtain the followings:

$$\begin{array}{l} L_{not\;linked}=\prod_{t}\sum_{\begin{array}{l} x_{1}(t)\\ x_{2}(t) \end{array}}P(x_{1}(t))\cdot P(x_{2}(t+\tau ))\cdot \frac{P(x_{1}(t)|y_{1}(t))\cdot P(y_{1}(t))}{P(x_{1}(t))}\cdot \frac{P(x_{2}(t+\tau )|y_{2}(t+\tau ))\cdot P(y_{2}(t))}{P(x_{2}(t+\tau ))}=\\ =\prod_{t}\sum_{\begin{array}{l} x_{1}(t)\\ x_{2}(t) \end{array}}P(x_{1}(t)|y_{1}(t))\cdot P(y_{1}(t))\cdot P(x_{2}(t+\tau )|y_{2}(t+\tau ))\cdot P(y_{2}(t))=\\ =\prod_{t}P(y_{1}(t))\cdot P(y_{2}(t))\sum_{\begin{array}{l} x_{1}(t)\\ x_{2}(t) \end{array}}P(x_{1}(t)|y_{1}(t))\cdot P(x_{2}(t+\tau )|y_{2}(t+\tau )), \end{array}$$

$$\begin{array}{l} L_{linked}=\prod_{t}\sum_{\begin{array}{l} x_{1}(t)\\ x_{2}(t) \end{array}}P(x_{1}(t))\cdot P(x_{2}(t+\tau )|x_{1}(t))\cdot \frac{P(x_{1}(t)|y_{1}(t))\cdot P(y_{1}(t))}{P(x_{1}(t))}\cdot \frac{P(x_{2}(t+\tau )|y_{2}(t+\tau ))\cdot P(y_{2}(t))}{P(x_{2}(t+\tau ))}=\\ =\prod_{t}\sum_{\begin{array}{l} x_{1}(t)\\ x_{2}(t) \end{array}}\frac{P(x_{2}(t+\tau )|x_{1}(t))}{P(x_{2}(t+\tau ))}\cdot P(x_{1}(t)|y_{1}(t))\cdot P(y_{1}(t))\cdot P(x_{2}(t+\tau )|y_{2}(t+\tau ))\cdot P(y_{2}(t))=\\ =\prod_{t}P(y_{1}(t))\cdot P(y_{2}(t))\sum_{\begin{array}{l} x_{1}(t)\\ x_{2}(t) \end{array}}\frac{P(x_{2}(t+\tau )|x_{1}(t))}{P(x_{2}(t+\tau ))}\cdot P(x_{1}(t)|y_{1}(t))\cdot P(x_{2}(t+\tau )|y_{2}(t+\tau )). \end{array}$$

The terms of *P*(*y*_1_(*t*)) and *P*(*y*_2_(*t*)) are taken outside of the sum in both formulas because *P*(*y*_*k*_(*t*)) is constant as *y*_*k*_(*t*) is the realization of random variable *x*_*k*_(*t*). They will be cancelled out in likelihood ratio and they can be omitted in formulas for *L*_*not linked*_ and *L*_*linked*_. So, the formulas be rewritten as next:

$$L_{not\;linked}\propto \prod_{t}\sum_{\begin{array}{l} x_{1}(t)\\ x_{2}(t) \end{array}}p(x_{1}(t)|y_{1}(t))\cdot P(x_{2}(t+\tau )|y_{2}(t+\tau )),$$ (4)

$$L_{linked}\propto \prod_{t}\sum_{\begin{array}{l} x_{1}(t)\\ x_{2}(t) \end{array}}\frac{P(x_{2}(t+\tau )|x_{1}(t))}{P(x_{2}(t+\tau ))}\cdot p(x_{1}(t)|y_{1}(t))\cdot P(x_{2}(t+\tau )|y_{2}(t+\tau )).$$ (5)

The estimate of conditional probability *P*(*x*_*k*_(*t*)|*y*_*k*_(*t*)) is the empirical cdf ${\hat{F}}_{k}(t)$ and $P(x_{k}(t)=1|y_{k}(t))={\hat{F}}_{k}(t),$, $P(x_{k}(t)=0|y_{k}(t))=1-{\hat{F}}_{k}(t).$

For simplicity, we will use $p_{x1x2}{}^{t}$ notation instead of product *P*(*x*_1_(*t*)|*y*_1_(*t*))‧*P*(*x*_2_(*t* + *τ*)|*y*_2_(*t* + *τ*)).

For example, *p*_00_^*t*^ = *P*(*x*_1_(*t*) = 0|*y*_1_(*t*))‧*P*(*x*_2_(*t* + *τ*) = 0|*y*_2_(*t* + *τ*)).

Note that *P*(*x*_*k*_(*t*)) is a marginal probability and it can be computed as follows:

$$q_{0k}=P(x_{k}(t)=0)=\frac{\sum_{t}P(x_{k}(t)=0|y(t))}{m},$$

$$q_{1k}=P(x_{k}(t)=1)=\frac{\sum_{t}P(x_{k}(t)=1|y(t))}{m}.$$

The conditional probability of Boolean state of variable *x*_2_(*t*+*τ*) given *x*_1_(*t*) in (5) becomes:

$$\begin{array}{l} P(x_{2}(t+\tau )|x_{1}(t))=\{\begin{array}{l} \delta (x_{2}(t+\tau )=f(x_{1}(t))),\text{if}\;f(x_{1}(t))\neq \Omega ;\\ P(x_{2}(t+\tau )),\text{if}\;f(x_{1}(t))=\Omega ; \end{array}\;\Omega =\{0\;\text{or}\;1\}\text{.} \end{array}$$ (6)

Eq (6) specifies the pattern for each of six possible Boolean functions of one variable. If it is f1 (equivalence), then *p*_00_ and *p*_11_ are given weight 1, while *p*_01_ and *p*_10_ are set to zero for the accordance to the truth table of f1. For computation reason in practice, we will use *ε* and 1−*ε* instead of 0 and 1 to avoid computing log(0) in log likelihood. The parameter *ε* can be adjusted if needed. Based on our empiric experience, it does not notably affect output. The default value of *ε* in software implementation is set to 0.005 in this study. However, the decrease of *ε* can slightly increase number of regulatory relations in output. For the functions which have indefinite output for one of inputs (f3-f6), we use the marginal probability of the second gene to be 1 or 0 as weight function. With all notations explained above, the likelihoods corresponding to all possible six functions between two genes can be written as follows:

$${\text{L}}_{\tau \text{,}f\text{1}}=\prod_{t}\left[\left(p_{00}{}^{t}/q_{02}+p_{11}{}^{t}/q_{12}\right)\cdot (1-\varepsilon )+(p_{01}{}^{t}/q_{12}+p_{10}{}^{t}/q_{02})\cdot \varepsilon \right],$$

$${\text{L}}_{\tau \text{,}f\text{2}}=\prod_{t}\left[\left(p_{01}{}^{t}/q_{12}+p_{10}{}^{t}/q_{02}\right)\cdot (1-\varepsilon )+(p_{00}{}^{t}/q_{02}+p_{11}{}^{t}/q_{12})\cdot \varepsilon \right],$$

$$\begin{array}{l} {\text{L}}_{\tau \text{,}f\text{3}}=\prod_{t}\left[p_{00}{}^{t}\cdot (1-\varepsilon )/q_{02}+p_{10}{}^{t}+p_{11}{}^{t}+p_{01}{}^{t}\cdot \varepsilon /q_{12}\right], \end{array}$$ (7)

$${\text{L}}_{\tau \text{,}f\text{4}}=\prod_{t}\left[p_{01}{}^{t}\cdot (1-\varepsilon )/q_{12}+p_{10}{}^{t}+p_{11}{}^{t}+p_{00}{}^{t}\cdot \varepsilon /q_{02}\right],$$

$${\text{L}}_{\tau \text{,}f\text{5}}=\prod_{t}\left[p_{10}{}^{t}\cdot (1-\varepsilon )/q_{02}+p_{00}{}^{t}+p_{01}{}^{t}+p_{11}{}^{t}\cdot \varepsilon /q_{12}\right],$$

$${\text{L}}_{\tau \text{,}f\text{6}}=\prod_{t}\left[p_{11}{}^{t}\cdot (1-\varepsilon )/q_{12}+p_{00}{}^{t}+p_{01}{}^{t}+p_{10}{}^{t}\cdot \varepsilon /q_{02}\right].$$

Similarly, ${\text{L}}_{\tau \text{,}not\;linked}=\prod_{t}\left[p_{11}{}^{t}+p_{00}{}^{t}+p_{10}{}^{t}+p_{01}{}^{t}\right].$

The largest of likelihoods *L*_*f*1_…*L*_*f*6_ will suggest the function $\hat{f}$ which is the best in explaining relation between two genes for given time delay *τ*. At the same time we need to find optimal time delay between genes. Thus we repeat procedure for all possible time delays and choose the one which corresponds to the largest difference in log-likelihoods of two models.

Significance of the established link is measured with p-value. Under the null hypothesis, the test statistic of 2 log(*R*) can be approximated by the Chi-square distribution.

In summary, the algorithm of link identification can be formulated as follows:

$$(x_{1},x_{2},\hat{f},\hat{\tau })=\text{arg}\underset{\tau_{i}}{\text{max}}(\underset{f(x_{1})}{\text{max}}l_{linked}-l_{no\_link}).$$

### Test 2

Fig 2 provides the graphical representation for two models: M0 assumes that there is intermediate gene *x*_3_ between genes *x*_1_ and *x*_2_; while in model M1 gene *x*_1_ regulates *x*_2_ directly. We assign *τ*′ to be time delay between *x*_1_ and *x*_3_, and *τ*″ to be time delay between *x*_3_ and *x*_3_. After the significant pairwise dependencies found by Test 1, Test 2 will test each link $\left(x_{1},x_{2},{\hat{f}}_{1-2},\hat{\tau }\right)$ such that links of $\left(x_{1},x_{3},{\hat{f}}_{1-3},{\hat{\tau }}^{\prime }\right)$ and $\left(x_{3},x_{2},{\hat{f}}_{3-2},{\hat{\tau }}^{\prime \prime }\right)$ exist and their time delays satisfy ***τ*′** + ***τ*″**≤ ***τ***.

The corresponding likelihoods of direct model M1 and indirect model M0 in Fig 2 can be expressed as next:

$$L_{0}=\prod_{t}\sum_{\begin{array}{l} x_{1}(t)\\ x_{3}(t+\tau^{\prime })\\ x_{2}(t+\tau ) \end{array}}\begin{array}{l} P(x_{1}(t))\cdot P(x_{3}(t+\tau^{\prime })|x_{1}(t))\cdot P(x_{2}(t+\tau )|x_{3}(t+\tau^{\prime }))\cdot \\ \cdot P(y_{1}(t)|x_{1}(t))\cdot P(y_{3}(t+\tau^{\prime })|x_{3}(t+\tau^{\prime }))\cdot P(y_{2}(t+\tau )|x_{2}(t+\tau )), \end{array}$$

$$L_{1}=\prod_{t}\sum_{\begin{array}{l} x_{1}(t)\\ x_{3}(t+\tau^{\prime })\\ x_{2}(t+\tau ) \end{array}}\begin{array}{l} P(x_{1}(t))\cdot P(x_{3}(t+\tau^{\prime })|x_{1}(t))\cdot P(x_{2}(t+\tau )|x_{3}(t+\tau^{\prime }),x_{1}(t))\cdot \\ \cdot P(y_{1}(t)|x_{1}(t))\cdot P(y_{3}(t+\tau^{\prime })|x_{3}(t+\tau^{\prime }))\cdot P(y_{2}(t+\tau )|x_{2}(t+\tau )). \end{array}$$

However, it is unnecessary to compute all parts since we are only interested in the difference, that is, the unary function of *f*(*x*_3_) against the binary function of *F*(*x*_1_, *x*_3_). Since the link *x*_1_→*x*_3_ is present in both models and it does not contribute to models differentiation, we can remove it from computation. Thus the corresponding likelihoods for two models can be written as next:

$$L_{0}=\underset{t}{\prod }\underset{\begin{array}{l} x_{3}(t+\tau^{\prime })\\ x_{2}(t+\tau ) \end{array}}{\sum }P(x_{3}(t+\tau \prime ))P(x_{2}(t+\tau )\left|x_{3}(t+\tau^{\prime }))\cdot P(y_{3}(t+\tau^{\prime })\right|x_{3}(t+\tau^{\prime }))\cdot P(y_{2}(t+\tau )|x_{2}(t+\tau )),$$

$$L_{1}=\underset{t}{\prod }\underset{\begin{array}{l} x_{1}(t)\\ x_{3}(t+\tau^{\prime })\\ x_{2}(t+\tau ) \end{array}}{\sum }\begin{array}{l} P(x_{1}(t))\cdot P(x_{3}(t+\tau \prime ))\cdot P(x_{2}(t+\tau )|x_{3}(t+\tau^{\prime }),x_{1}(t))\cdot \\ \cdot P(y_{1}(t)\left|x_{1}(t))\cdot P(y_{3}(t+\tau^{\prime })\right|x_{3}(t+\tau^{\prime }))\cdot P(y_{2}(t+\tau )|x_{2}(t+\tau )). \end{array}$$

After applying Bayes’ theorem and all reductions similar to Test 1, the likelihood of indirect model M0 and direct model M1 can be written as follows:

$$L_{0}=\prod_{t}\sum_{\begin{array}{l} x_{3}(t+\tau \prime )\\ x_{2}(t+\tau ) \end{array}}\frac{P(x_{2}(t+\tau )|x_{3}(t+\tau \prime ))}{P(x_{2}(t+\tau ))}\cdot p(x_{3}(t+\tau \prime )|y_{3}(t+\tau \prime ))\cdot P(x_{2}(t+\tau )|y_{2}(t+\tau )),$$

$$L_{0}=\prod_{t}\sum_{\begin{array}{l} x_{1}(t)\\ x_{3}(t+\tau \prime )\\ x_{2}(t+\tau ) \end{array}}\frac{P(x_{2}(t+\tau )|x_{3}(t+\tau \prime ),x_{1}(t))}{P(x_{2}(t+\tau ))}\cdot p(x_{1}(t)|y_{1}(t))\cdot P(x_{3}(t+\tau \prime )|y_{3}(t+\tau \prime ))\cdot P(x_{2}(t+\tau )|y_{2}(t+\tau )).$$

Similarly to (6), we have

$$P(x_{2}(t+\tau )|x_{1}(t),x_{3}(t+\tau \prime ))=\left\{ \begin{array}{l} \delta [x_{2}(t+\tau )=F(x_{1}(t),x_{3}(t+\tau \prime ))],\;\text{if}\;F(x_{1}(t),x_{3}(t+\tau \prime ))\neq \Omega ;\\ P(x_{2}(t+\tau )),\;\text{if}\;F(x_{1}(t),x_{3}(t+\tau \prime ))=\Omega ;\; \end{array}\right.\;\Omega =\{0\;\text{or}\;1\}\text{.}$$

Analogously to (7), we can have some examples of the formulas for above mentioned likelihoods as follows:

$$L_{\tau ,\tau \prime ,F1}=\prod_{t}\left[\begin{array}{l} (p_{000}{}^{t}/q_{02}+p_{010}{}^{t}/q_{02}+p_{100}{}^{t}/q_{02}+p_{111}{}^{t}/q_{12})(1-\varepsilon )+\\ +(p_{001}{}^{t}/q_{12}+p_{011}{}^{t}/q_{12}+p_{101}{}^{t}/q_{12}+p_{110}{}^{t}/q_{02})\varepsilon \end{array}\right],$$

$$L_{\tau ,\tau \prime ,F11}=\prod_{t}\left[\begin{array}{l} (p_{010}{}^{t}/q_{02}+p_{100}{}^{t}/q_{02}+p_{110}{}^{t}/q_{02})(1-\varepsilon )+p_{000}+p_{001}+\\ +(p_{011}{}^{t}/q_{12}+p_{101}{}^{t}/q_{12}+p_{111}{}^{t}/q_{12})\varepsilon \end{array}\right].$$

Among all candidates, we choose such intermediate gene in model M1 which can maximize likelihood of this model and therefore maximize difference between models. In the last step, we need to make sure that we choose optimal time delay ***τ*″** between intermediate gene *x*_3_ and target gene *x*_2_. Thus we select ***τ*′** such that the largest likelihood ratio *R*_*x*3_(*τ*, *τ*′) refers to the best choice of *x*_3_.

On the whole, the Test 2 procedure can be expressed as next:

$$(x_{1},x_{3},x_{2},\hat{F},\hat{\tau },\hat{\tau^{\prime }})=\text{arg}\underset{\tau^{\prime }}{\text{max}}\underset{x_{3}}{\text{max}}(\underset{F(x_{1},x_{3})}{\text{max}}l_{1}-\underset{f(x_{3})}{\text{max}}l_{0}).$$

### Complexity of the algorithm

Boolean networks can be very useful in finding dependencies among genes. However the exhaustive search of the optimal Boolean network is infeasible for the study of a large number of genes. The proposed BFN algorithm has the computational complexity of *O*(*n*^3^) in worst case when the GRN is a complete directed graph. In Test 1, we consider *n* (*n–* 1) possible gene pairs. For every gene pair which passed Test 1, we consider at most (*n–* 2) intermediate genes in Test 2. However, these two tests are conducted in sequence, not in a nest loop. This allows the reduction of computational complexity significantly because there are only a limited number of gene pairs that will pass Test 1 and enter Test 2.

## Supporting information

S1 Table Whole-genome *S*. *cerevisiae* GRN inferred with BFN method. (XLSX)

S2 Table Detailed description of BFN performance measured with reference to SGD, Gold-standard GRN #1, Gold-standard GRN #2, Gold-standard GRN #3. (XLSX)

S3 Table Detailed description of BFN (Test1) performance measured with varying p-values on set of 103 cell-cycled genes. (XLSX)

S4 Table Positive links with cycle phase annotation inferred with BFN on set of 103 cell-cycled genes. (XLSX)

S5 Table Gene ontology (GO) and pathway enrichment for groups of target genes. (XLSX)

S6 Table Subnetwork for 9 glycolytic genes inferred with BFN. (XLSX)

S7 Table Source/target annotation for whole genome and 103 cell-cycled genes experiments; cycle phase annotation for 103 genes. (XLSX)
