# Correction: Exploring candidate biological functions by Boolean Function Networks for *Saccharomyces cerevisiae*

**DOI:** 10.1371/journal.pone.0221703

**Published:** 2019-08-22

**Authors:** Maria Simak, Chen-Hsiang Yeang, Henry Horng-Shing Lu

An affiliation for the first author is not indicated. Maria Simak is also affiliated with: Institute of Bioinformatics and Systems Biology, National Chiao Tung University, Hsinchu, Taiwan.

## References

[1] Simak M, Yeang C-H, Lu HH-S (2017) Exploring candidate biological functions by Boolean Function Networks for *Saccharomyces cerevisiae*. PLoS ONE 12(10): e0185475 10.1371/journal.pone.0185475 28981547 PMC5628832

